# 以肺性肥大性骨关节病为首发表现的肺癌1例报道

**DOI:** 10.3779/j.issn.1009-3419.2010.09.15

**Published:** 2010-09-20

**Authors:** 林娟 连, 殿胜 钟, 松 吴

**Affiliations:** 300052 天津，天津医科大学总医院呼吸科 Department of Respiratory Medicine, Tianjin Medical University General Hospital, Tianjin 300052, China

## 1 病例资料

患者，男，54岁，因“指(趾)末端无痛性、进行性增粗3年，膝、踝关节疼痛伴活动障碍2年”入住天津医科大学总医院。患者曾先后就诊于内分泌科和血液科，未明确诊断。自发病以来，无咳嗽、咳痰、咯血、胸痛等症状。既往体健，吸烟40年，20支/天。其母死于肺癌。入院查体：甲床无紫绀；双肺呼吸音清，未闻及啰音；心音有力，律齐，未闻及杂音；明显杵状指(趾)伴周围皮肤红肿、发亮，皮温稍高，无触痛，呈对称分布(图 1)；双侧膝、踝关节轻度肿胀，轻压痛，浮髌试验阴性。实验室检查：血、尿、便常规、凝血功能、肝肾功能、电解质以及免疫全项(抗核抗体、类风湿因子和抗中性粒细胞胞浆抗体)等均正常。双膝X线示：双侧股骨下端、胫骨上端及腓骨近端可见对称性骨膜增生，考虑双膝关节肺性肥大性骨关节病(图 2)。全身骨骼单电子发射计算机断层成像术(single-photon emission computed tomography, SPECT)骨显像示：双侧股骨、胫骨皮质对称性摄取核素能力增强，以双侧胫骨尤为明显，呈现“双轨征”(double stripe)(图 3)，符合肺性肥大性骨关节病图像。胸部薄层CT及增强显示：右肺上叶尖段软组织密度肿块影，大小约3.3 cm×3.0 cm×2.6 cm，呈分叶状，周围可见毛刺，与胸膜粘连，增强后不均匀强化；右肺上叶胸膜下可见一空洞性病变，直径约1.0 cm，其内壁不光滑，外壁毛糙。考虑为肿瘤性病变(图 4)。纤维支气管镜提示支气管慢性炎症，于右肺上叶尖段取肺活检，病理回报为“细支气管肺泡癌”。肺功能正常，腹部及头颅CT等正常。综上所述，临床诊断：右上叶周围型肺癌，临床分期T3N0M0，IIb期，肺性肥大性骨关节病。后转入胸外科，于全麻下行右肺上叶切除及系统淋巴结清扫。术后病理回报：中分化腺癌，部分呈微乳头腺癌，部分呈细支气管肺泡癌，侵及胸膜，余无转移。术后1周，关节疼痛明显缓解。先后行4个疗程的GP方案(吉西他滨+顺铂)化疗，杵状指(趾)有所缓解，周围皮肤红肿基本消失。

**1:**
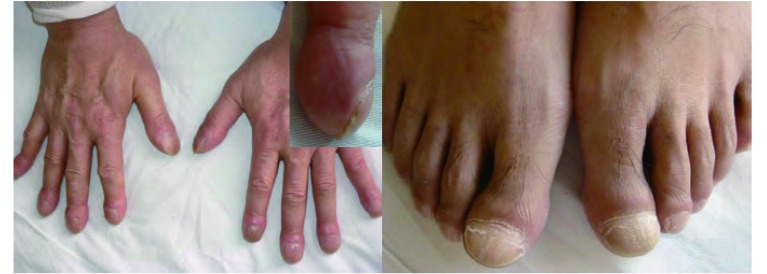
明显杵状指(趾)伴周围皮肤红肿、发亮，呈对称分布。 Significantly clubbing digits accompanied with the periphery skin being flush and flashing, symmetricly.

**2:**
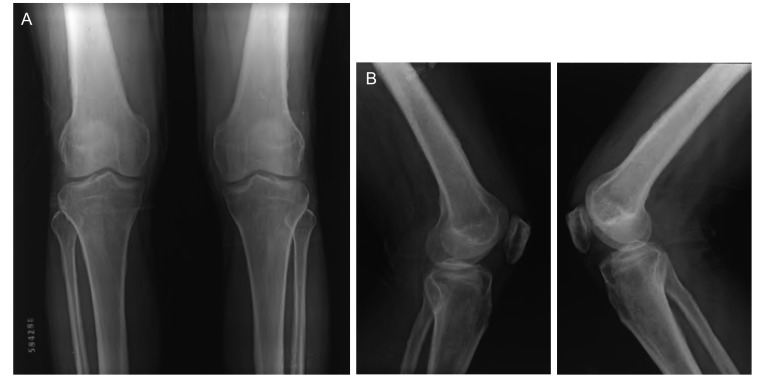
双膝关节X线检查。双侧股骨远端、胫骨上端及腓骨近端可见对称性骨膜增生。A：正位；B：侧位。 X-ray of both knees. The distal part of bilateral femur and the proximal part of tibiaand fibula show symmetry periosteal proliferation. A: normotopia; B: lateral.

**3:**
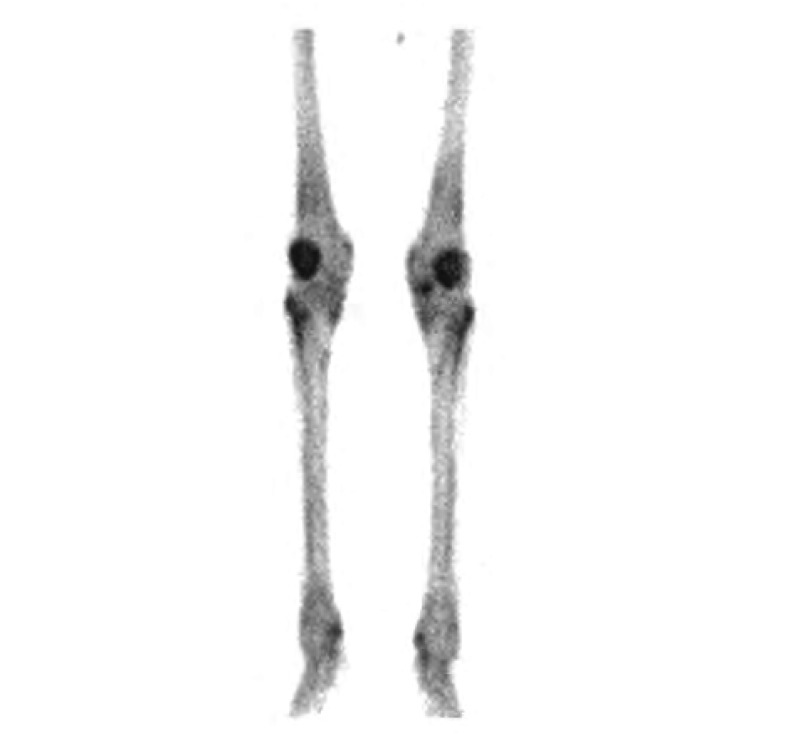
SPECT骨显像：双侧股骨、胫骨皮质对称性摄取核素能力增强，呈“双轨征”。 SPECT bone imaging: the cortical bone of bilateral femur and tibia show more nuclide uptaking, "double stripe".

**4:**
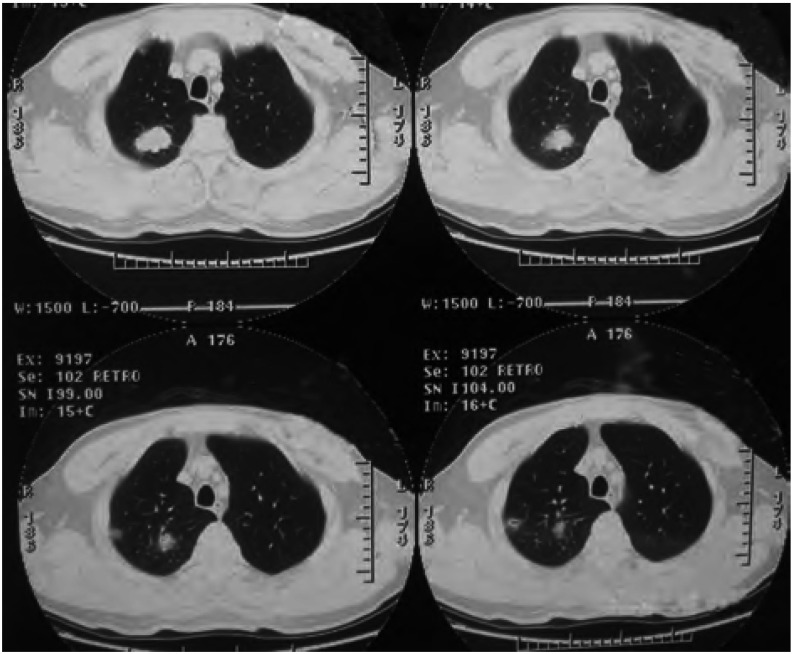
胸部CT。右肺上叶尖段可见3.3 cm×3.0 cm×2.6 cm软组织密度影，呈分叶状，周围可见毛刺；右肺上叶胸膜下可见一空洞性病变，直径约1.0 cm，其内壁不光滑，外壁毛糙。 Chest CT shows a lobulated parenchyma density shadow with glitch at the apical segment of the superior lobe of right lung, 3.3 cm ×3.0 cm×2.6 cm; another lesion has a cavity, the diameter of the cavity is 1.0 cm, with coarse intine and exine.

## 2 讨论

肺性肥大性骨关节病(pulmonary hypertrophic osteoarthropathy, PHOA)由Marie和Bamberger于19世纪后期首先报道，故又称Marie-Bamberger综合征，是以对称性四肢管状骨骨膜广泛增生、杵状指(趾)和关节炎为特征的骨关节病变，其特征表现包括：①骨膜下新骨形成(主要沿长骨生长)；②对称性关节病；③杵状指(趾)；④长骨、指(趾)、鼻、偶尔耳廓周围组织血流增多；⑤与恶性肿瘤关系密切^[1-3]^。

PHOA可分为原发性和继发性两种。原发性PHOA十分罕见，临床上以继发性为主，其中约80%-90%继发于胸内疾患，故称之为肺性肥大性骨关节病。在胸内疾患中，80%由肺癌引起，综合文献所述，以鳞癌和腺癌多见，右侧多于左侧，上叶多于下叶，周围型多于中央型；也可见于肺转移瘤和胸膜肿瘤，偶见于儿童肿瘤(如鼻咽癌和淋巴瘤)。良性慢性肺部疾病，如肺脓肿、慢性支气管炎、肺结核、肺炎、支气管扩张、胸膜炎等也有引起PHOA的报道。

肺癌患者并发PHOA的发生率约0.7%-12%^[4]^。美国Mayo Clinic报道的1 872例肺部肿瘤切除的患者中，10%患有骨关节病变^[5]^。陶宝华等^[6]^报道671例肺癌患者的骨显像中，PHOA发生率为7.6%，考虑到病例的来源(核医学科)，实际发病率应该没有这么高，但PHOA也并非肺癌的少见副癌综合征表现。因为，绝大多数该类患者以骨关节病变和杵状指(趾)为首发症状，经常误诊为风湿或类风湿性关节炎，确诊时间3个月-14个月不等^[7]^。本例患者先后就诊于内分泌科和血液科，确诊时间长达3年。

骨显像对于本病的诊断和鉴别诊断具有极其重要的价值^[6, 8, 9]^，因为肺癌患者发生骨痛可以是骨转移也可以是PHOA。PHOA骨显像图的典型表现是沿着长骨内、外侧缘呈平行放射性摄取，称为“双轨征”，主要发生在四肢长骨骨干等缺少红骨髓的部位，规律、对称性弥漫性异常的放射性增强，很少累及中轴骨；而肺癌主要通过血行转移至红骨髓丰富的骨骼，如中轴骨(脊柱、肋骨和骨盆等)，且多呈不规则性、局限性、非对称性放射性浓集。此外，骨显像异常经常早于临床症状和/或X线异常。

当原发病灶被控制或治愈后，本病的关节症状-杵状指(趾)均可减退或消失，增生的骨膜可逐渐吸收；研究^[10]^显示，肿瘤切除或放、化疗后，37%的患者疼痛立即减轻或消失，84%患者术后1个月疼痛消失，骨膜增生在肺部病变治愈后2个月-3个月逐渐消退，杵状指(趾)消失缓慢，肿瘤复发时，症状复现。本例患者术后1周，关节疼痛明显缓解。此外，杵状指(趾)逐渐缓解。

为了减少本病的误诊，临床上对于原因不明的杵状指(趾)，尤其是合并骨关节肿胀、疼痛并呈进行性肥大的患者，一定要警惕PHOA，并进行胸部X线检查除外肺癌。需要指出的是，PHOA是肺癌的一个非常重要的非转移性肺外表现，但决不仅见于肺癌。
